# New Analytical Screening Method for Fast Classification of Hemp Oil Based on THC Content

**DOI:** 10.1021/acsomega.4c10753

**Published:** 2025-04-08

**Authors:** Thaineh E. A. Souza, Gustavo Bertol, Poliana M Santos

**Affiliations:** † Universidade Tecnológica Federal do Paraná, 81280-340 Curitiba, Paraná, Brazil; ‡ Dall PhytoLab SA, 82540-040 Curitiba, Paraná, Brazil

## Abstract

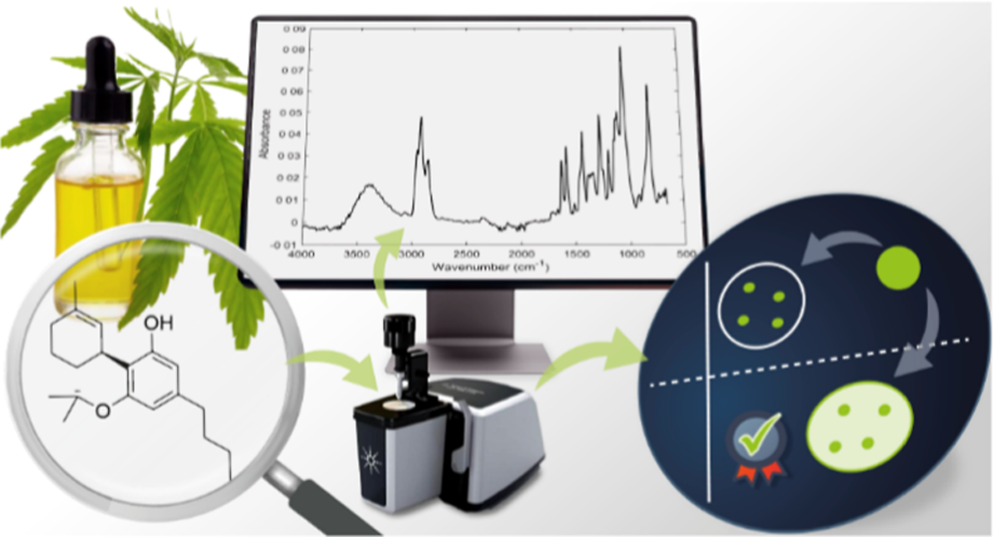

This study presents a novel analytical approach for classifying commercial hemp oil samples according to their Δ9-tetrahydrocannabinol (THC) content, employing mid-infrared (MIR) spectroscopy combined with machine learning algorithms. A total of 204 commercial hemp oil samples, with THC concentrations ranging from 0.0% to 16.6% w/w, were analyzed. Partial least-squares-discriminant analysis (PLS-DA) was employed for classification purposes. Two classification models were developed based on international regulatory thresholds: model A, which classifies samples with THC concentrations exceeding 0.2% w/w, and model B, designed to classify those with THC levels above 0.3% w/w. Both models demonstrated good performance, achieving accuracy values higher than 88.50%. Notably, model B reduced false negatives, improving sensitivity (STR) values from 93.75% to 98.31% for the training set and from 77.27% to 95.00% for the test set, compared to model A. This approach offers a viable alternative to conventional laboratory methods by eliminating complex sample preparation steps and enabling simple and rapid THC screening.

## 1 Introduction

**Figure 1:**
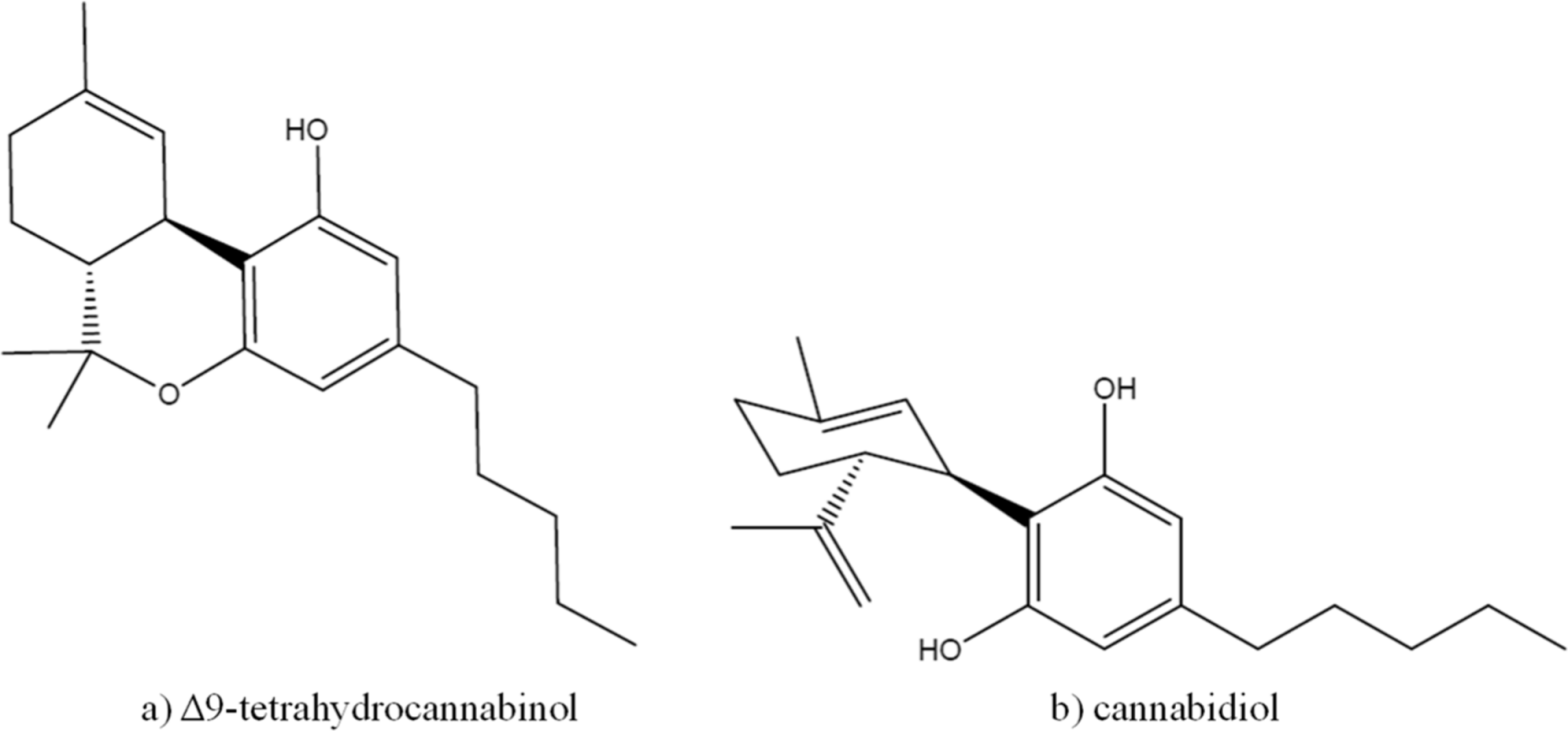
Chemical structures of (a) THC and (b) CBD cannabinoids.

In recent years, the use of cannabis-based products for medicinal purposes has increased significantly due to their therapeutic benefits. Derived from the well-known *Cannabis sativa* L. plant, which contains at least 554 identified compounds, including more than 100 cannabinoids,^1^ their potential health benefits are mainly attributed to the pharmacological properties of the cannabinoids Δ9-tetrahydrocannabinol (THC) and cannabidiol (CBD).^2^ THC, known for its psychoactive effects, has shown potential in stimulating appetite in patients with cancer and HIV/AIDS and in reducing nausea and vomiting, particularly in those undergoing chemotherapy.^3^ In contrast, the nonpsychoactive CBD has demonstrated anticonvulsive, anti-inflammatory, analgesic, and neuroprotective properties.^3−5^Figure 1 illustrates the chemical structures of the cannabinoids THC and CBD. Both compounds have the same molecular formula, C_21_H_30_O_2_, and consist of a cyclohexene ring, a phenolic ring, and a pentyl side chain. Despite these similarities, THC contains a cyclic ether ring, whereas CBD features a hydroxyl group. This subtle difference in molecular structure underlies their distinct pharmacological properties.^6^

Currently, a wide range of cannabis-based products are available on the market. Consequently, many countries have implemented specific legislation to regulate their production and distribution. These legislations include limits on THC content. Ensuring that cannabis-derived products meet these regulatory thresholds is essential, not only for legal compliance but also for consumer safety, as THC levels directly affect the psychoactive effects of these products. Additionally, maintaining appropriate THC levels helps direct the product toward the desired clinical applications, considering the distinct pharmacological actions of CBD and THC. In the European Union, the lowest limit is typically set at 0.2% w/w,^7,8^ whereas in the USA, it is set at 0.3% w/w.^9^ In Brazil, the National Health Surveillance Agency (ANVISA) has established that THC content must not exceed 0.2% w/w.^10^ However, an exception applies to products intended for palliative care in patients with irreversible or terminal conditions, where other therapeutic options are not viable.^10^

Liquid chromatography,^11−13^ alongside gas chromatography,^14,15^ has been the preferred technique for quantifying THC in cannabis-based products. However, these methods often require time-consuming sample preparation procedures and involve the use of large amounts of organic solvents, expensive instruments, and analytical standards, making them less feasible for large-scale or in situ applications. As the demand for cannabis products continues to grow, alternative analytical methods have been developed to address these limitations, focusing on speed, environmental sustainability, and cost-effectiveness.^16−21^

In this context, infrared spectroscopy, particularly in the mid-infrared (MIR) and near-infrared (NIR) regions, has gained attention as a promising alternative for the rapid and nondestructive analysis of cannabis-based products. These techniques enable direct analysis without extensive sample preparation and can be easily adapted for in situ analysis using portable equipment. Previous studies have successfully employed infrared spectroscopy to quantify THC in fluids,^22^ hemp oil,^23,24^ cannabis flowers, and extracts.^8,25^ However, despite these advancements, there is still a lack of simple screening methods for the rapid classification of commercial hemp oil based on THC content. The methods reported in the literature have concentrated on analyzing the cannabis plant itself, often aiming to classify it according to growth stage,^26^ chemovar,^27^ or fiber type.^28^ In this sense, a practical and cost-effective approach for fast screening of THC content has not been proposed yet.

Recently, Monari et al. (2025) investigated the use of an electrochemical sensor to recognize extracts of *Cannabis sativa* L. based on THC content.^29^ Classification models were developed using the partial least-squares-discriminant analysis (PLS-DA) method, which successfully classified the samples into legal and illegal categories, with threshold limits set at 0.3% and 0.6% w/w. However, it is important to highlight that the study focused on cannabis extracts. Therefore, developing new strategies to facilitate and improve the control of commercial cannabis-based products is crucial, considering the potential impact of THC on health.

In this context, the present study pioneeringly proposes the use of MIR spectroscopy combined with the PLS-DA method as an alternative approach for the rapid qualitative discrimination of commercial hemp oil samples based on their THC content. The models were validated by estimating the sensitivity rate (STR), specificity rate (SPR), and accuracy figures of merit. The advantages of the proposed method include speed, simplicity, cost-effectiveness, and the elimination of sample preparation requirements.

## 2 Materials and Methods

### 2.1 Samples

This study analyzed a total of 204 commercial hemp oil samples with THC concentrations spanning from 0.0% to 16.6% w/w. The overall sample set comprised full-spectrum and CBD/THC isolated products from a wide variety of manufacturers from different countries (Brazil, United States, Switzerland, and Colombia). THC concentrations were determined using high-performance liquid chromatography with diode-array detection (HPLC–DAD). Analyses were conducted on an Agilent 1260 Infinity HPLC system (Agilent Technologies Inc.), with the DAD set to 228 nm. Separation was achieved using a C18 chromatographic column (150 × 4.6 mm, 2.7 μm). The mobile phase consists of trifluoroacetic acid in acetonitrile, mixed in a 41:59 (v/v) ratio. The trifluoroacetic acid solution was prepared at 1% (v/v) by diluting the stock solution in purified water (resistivity >18 MΩ·cm).

### 2.2 Mid-Infrared Spectroscopy

The spectral measurements were performed using a Cary 630 Fourier transform infrared (FTIR) spectrometer (Agilent Technologies Inc.), equipped with an attenuated total reflection (ATR) module. The spectra were collected in the range of 4000–650 cm^–1^, with a resolution of 4 cm^–1^, averaging a total of 16 scans.

### 2.3 Data Analysis

PLS-DA was performed using MATLAB 2021a (MathWorks, Natick, USA), supported by the PLS_Toolbox 8.9.2 (Eigenvectors Research Inc., Manson, USA). Prior to the analysis, the data were preprocessed using the first derivative (Savitzky–Golay with a second-order polynomial and a 15-point window), followed by mean centering.

Considering global regulatory standards, PLS-DA models were developed with two different classification purposes. The first approach focused on distinguishing hemp oil samples with THC concentrations greater than 0.2% w/w (model A), whereas the second approach aimed to distinguish hemp oil samples with THC concentrations exceeding 0.3% w/w (model B). The Kennard–Stone algorithm was used to split the data into training and test sets, with 75% of the samples allocated to the training set and the remaining 25% to the test set. The number of latent variables (LV) was chosen by random subsets cross-validation (6 splits and 10 iterations), based on the smallest number of classification errors.

The assessment of the classification models was performed using sensitivity rate (STR), specificity rate (SPR), and accuracy. STR is defined as the ratio between true positives (TP) and the sum of TP and false negatives (FN), while SPR is the ratio between true negatives (TN) and the sum of TN and false positives (FP). Accuracy is the ratio of the sum of TP and TN to the total sum of TP, TN, FP, and FN. TP represents the number of correct predictions where the model accurately identified the positive class, i.e., samples with THC concentrations above the threshold (0.2% or 0.3% w/w) classified as positive. Similarly, TN denotes the number of correct predictions for the negative class, i.e., samples with THC concentrations below the threshold classified as negative. FP refers to samples that do not belong to the positive class but were incorrectly classified as such, i.e., samples with THC concentrations below the threshold misclassified as positive. Conversely, FN indicates samples that belong to the positive class but were misclassified as negative, i.e., samples with THC concentrations above the threshold incorrectly identified as negative.

## 3 Results and Discussion

### 3.1 MIR Spectra

**Figure 2:**
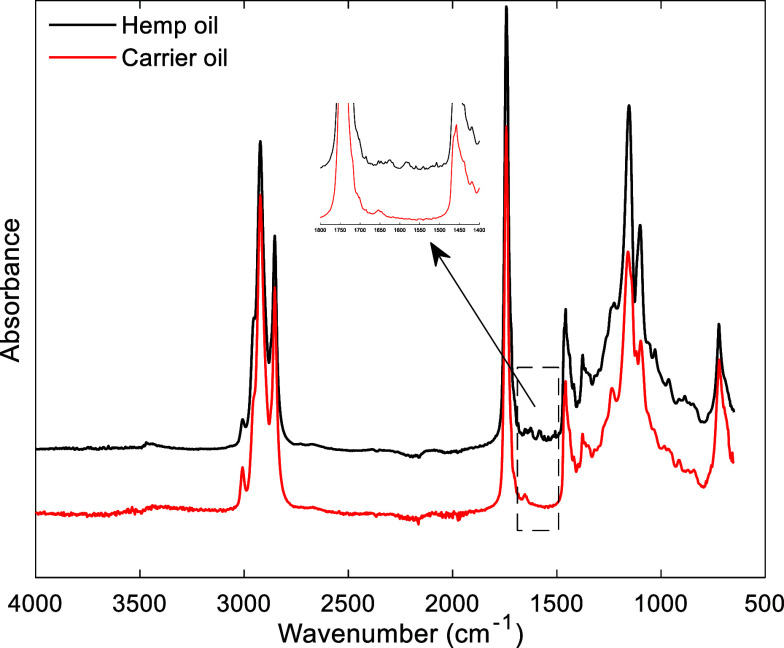
Average MIR spectrum of the 204 hemp oil samples and the carrier oil.

Figure 2 shows the average MIR spectrum of the 204 hemp oil samples and the carrier oil. Hemp oil is a common product derived from *Cannabis sativa* L. plants, whereas carrier oil is a vegetable oil used to dilute the *Cannabis sativa* L. extract. Many different oils can be used as carrier oils, with medium-chain triglyceride (MCT) being one of the most used. The general structure of MCT (Figure S1, Supporting Information) consists of three saturated fatty acids attached to a glycerol backbone. The R groups represent fatty acid molecules with chain lengths ranging from six to 12 carbon atoms.^30^

Analysis of the results indicates that the dominant signals observed in the MIR spectrum of hemp oil samples are primarily attributed to the components of the carrier oil. The strong absorptions at 2924 and 2855 cm^–1^ are associated with asymmetrical and symmetrical stretching modes of CH_2_ and CH_3_ of methylene groups. The band at 1740 cm^–1^ is attributed to the C=O stretching mode of ester carbonyls, whereas the bands at 1460 and 1377 cm^–1^ are associated with C–H bending vibrations of CH_2_ and CH_3_ in aliphatic groups. The bands in the 1151–1030 cm^–1^ region are attributed to the C–O stretching vibration of acyl ester groups. The weak absorption bands at 983 and 887 cm^–1^ have been associated with C–H bending vibrations and =CH_2_ wagging vibrations, respectively. In addition, the band at 725 cm^–1^ is related to the overlapping of CH_2_ rocking vibrations and out-of-plane vibration.

Although the spectra of hemp oil samples and the carrier oil are very similar, some important differences were identified, such as the small bands around 1621 and 1579 cm^–1^, which correspond to the C=C stretching vibration of aromatic rings. This signal also appears in the MIR spectrum of the THC cannabinoid standard shown in Figure S2 (Supporting Information). The THC cannabinoid standard spectrum was measured by placing 5 μL of a 1000 mg L^–1^ standard solution on the ATR crystal surface and allowing the solvent to evaporate in airflow. The MIR spectrum of the THC standard (Figure S2, Supporting Information) displays additional prominent signals in the fingerprint region (1600–800 cm^–1^), attributed to C=O stretching, C=C stretching vibrations, C–H bending, C–O–H in-plane bending, and C–O stretching vibrations. Additionally, the band between 3700 and 3100 cm^–1^ corresponds to the O–H stretching modes of the solvent.

### 3.2 PLS-DA Model Analysis

#### 3.2.1 Model A

Considering the regulatory standards in Brazil and some European countries, PLS-DA models were developed to target samples with THC concentrations exceeding 0.2% w/w. In this approach, samples with THC ≤ 0.2% w/w were assigned a *y*-value of 0.0, while those with THC > 0.2% w/w were assigned a *y*-value of 1.0. The training set comprised 152 hemp oil samples (88 samples with THC concentrations ≤ 0.2% w/w and 64 samples with THC concentrations > 0.2% w/w), while the test set consisted of 52 samples (30 samples with THC concentrations ≤ 0.2% w/w and 22 samples with THC concentrations > 0.2% w/w).

The PLS-DA model was built with 5 LV, accounting for 99.04% of the spectral variance (*X* block) and 75.64% of the class variance (*y* block). The model results are presented in the form of a confusion matrix (Table 1). In the training set, 87 out of 88 commercial hemp oil samples were correctly classified as THC concentrations ≤ 0.2% w/w, while 60 out of 64 samples were correctly identified as THC concentrations > 0.2% w/w. However, the model produced one FP, misclassifying one commercial hemp oil sample with THC ≤ 0.2% w/w as THC > 0.2% w/w, and four FN, misclassifying four samples with THC > 0.2% w/w as THC ≤ 0.2% w/w. For the test set, all commercial hemp oil samples with THC concentrations ≤ 0.2% w/w were correctly classified, while 17 out of 22 samples with THC concentrations >0.2% w/w were correctly identified, resulting in five FN. Among the nine FN (four in the training set and five in the test set), seven samples had THC levels between 0.21% and 0.29% w/w, which is notably close to the established class threshold. The remaining two samples exhibited THC levels of 0.41% and 0.54% w/w.

**Table 1:** Confusion Matrix of Training and Test Sets of Model A

	actual classes	
predicted classes	THC ≤ 0.2% w/w	THC > 0.2% w/w
Training		
THC ≤ 0.2% w/w	87	4
THC > 0.2% w/w	1	60
Test		
THC ≤ 0.2% w/w	30	5
THC > 0.2% w/w	0	17

The qualitative figure of merit for the model is shown in Table 2. Overall, the model demonstrated good performance, achieving a SPR of 98.86% and 100.00% for the training and test sets, respectively, indicating its effectiveness in accurately identifying TN samples. The STR, which measures the model’s ability to correctly identify TP samples, was 93.75% for the training set (60 out of 64 samples correctly classified). For the test set, the STR was 77.27% (17 out of 22 samples correctly classified). Model A achieved an accuracy exceeding 88%.

**Table 2:** Performance Parameters for the PLS-DA Classification Models Developed to Discriminate Hemp Oil Samples with THC Concentrations Greater Than 0.2% w/w (Model A) and 0.3% w/w (Model B)^a^

	model A		model B	
	training set	test set	training set	test set
STR (%)	93.75	77.27	98.31	95.00
SPR (%)	98.86	100.00	98.94	100.00
accuracy (%)	96.50	88.50	98.62	97.50

a Note: (STR) sensitivity rate; (SPR) specificity rate.

The variable importance in the projection (VIP) scores graph (Figure S3, Supporting Information) indicated that the most relevant bands for sample discrimination are found in the fingerprint region (650–1800 cm^–1^). Another important region spans from 1700 to 1000 cm^–1^, associated with C=O, C=C, and C–O stretching vibrations, as well as C–H bending vibrations. Although the signal around 2100 cm^–1^ has a VIP score above 1, it is linked to the diamond crystal of the ATR accessory and does not provide meaningful information. The regression vector (Figure S4, Supporting Information) revealed that the most important wavenumbers for class 0 (hemp oil samples with THC ≤ 0.2% w/w) are 1735, 1608, 1561, 1412, and 1036 cm^–1^. For class 1 (hemp oil samples with THC > 0.2% w/w), the most important wavenumbers are 1714, 1630, 1585, 1436, 1192, and 1056 cm^–1^.

#### 3.2.2 Model B

In the second approach, in compliance with USA legislation, the THC concentration threshold was established at 0.3% w/w. Accordingly, samples with THC levels of ≤ 0.3% w/w were assigned a *y*-value of 0.0, while those with THC levels >0.3% w/w received a *y*-value of 1.0. The training set comprised 153 hemp oil samples, with 94 samples having THC concentrations of ≤ 0.3% w/w and 59 samples with concentrations >0.3% w/w. The test set consisted of 51 hemp oil samples, with 31 samples having THC concentrations of ≤ 0.3% w/w and 20 samples with concentrations >0.3% w/w.

The final model was constructed with 5 LVs, explaining 98.84% and 82.04% of the variance in the *X* and *y* blocks, respectively. As shown in Table 3, one FP (a commercial hemp oil sample with THC concentration ≤ 0.3% w/w incorrectly classified as THC concentration >0.3% w/w) and one FN (a commercial hemp oil sample with THC concentration >0.3% w/w incorrectly classified as THC concentration ≤ 0.3% w/w) were observed during the training phase. These errors resulted in SPR and STR values of 98.94% and 98.31%, respectively. During the test phase, only one FN was observed, leading to SPR and STR values of 100.00% and 95.00%, respectively. The FN samples had a THC concentration of 0.41% and 0.54%. The qualitative figure of merit of the model is summarized in Table 2.

**Table 3:** Confusion Matrix of Training and Test Sets of Model A

	actual classes	
predicted classes	THC ≤ 0.3% w/w	THC > 0.3% w/w
Training		
THC ≤ 0.3% w/w	93	1
THC > 0.3% w/w	1	58
Test		
THC ≤ 0.3% w/w	31	1
THC > 0.3% w/w	0	19

The VIP scores graph (Figure S5, Supporting Information) showed a profile very similar to that of model A, with the most relevant signals located in the fingerprint region (650–1800 cm^–1^).

When comparing the performance of the models, a significant reduction in false negative samples was achieved in model B. In the training set, the number of false negatives decreased from four to one, while in the test set, it decreased from five to one. As a result, the STR values increased from 93.75% to 98.31% for the training set and from 77.27% to 95.00% for the test set. Accordingly, the overall accuracy improved from 96.50% to 98.62% for the training set and from 88.50% to 97.50% for the test set. This improvement is attributed to the simple strategy of avoiding false negatives by raising the threshold.

## 4 Conclusions

This study successfully demonstrates, for the first time, the feasibility of combining MIR spectroscopy with PLS-DA for the qualitative discrimination of commercial hemp oil samples based on their THC content. The proposed method is simple, fast, and cost-effective, enabling direct analysis without the need for sample preparation, thereby reducing waste generation. Additionally, the use of portable equipment facilitates in situ screening, allowing the identification of potentially noncompliant samples for further confirmatory analysis.

The classification models showed good accuracy, although their performance may be affected when THC levels are very close to the threshold, particularly at 0.2% (w/w). However, it is important to emphasize that the majority of regulations worldwide permit THC concentrations above 0.3%, and in these cases, the model performs exceptionally well, making it a valuable tool for rapid screening and compliance verification.

## Supporting Information Available

General structure of medium-chain triacylglycerol, MIR spectrum of THC standard solution, VIP scores, and regression vector graphics (PDF)
