# Supplementary material for: New Analytical Screening Method for Fast Classification of Hemp Oil Based on THC Content
Source: ACS Omega. 2025 Apr 8;10(15):15143–7. doi: 10.1021/acsomega.4c10753 (PMC12019721; doi:10.1021/acsomega.4c10753)
Supplement: Supplementary file 1 — ao4c10753_si_001.pdf [file ao4c10753_si_001.pdf]

Supplementary Materials

A new analytical screening method for fast classification of hemp oil based on THC content

Thaineh Emily Alves de Souza[a,b], Gustavo Bertol[b], Poliana M. Santos[a*]

[a]Universidade Tecnológica Federal do Paraná, 81280-340, Curitiba – PR, Brazil
[b]Dall PhytoLab SA, 82540-040, Curitiba – PR, Brazil

*Corresponding author
e-mail: polianasantos@utfpr.edu.br

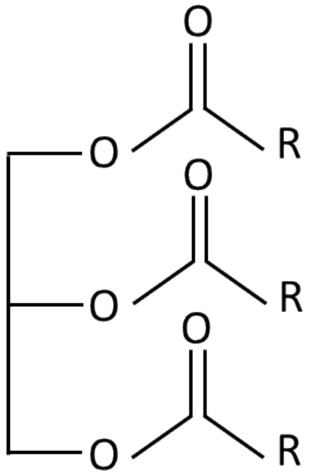

Fig. S1. General structure of medium-chain triacylglycerol

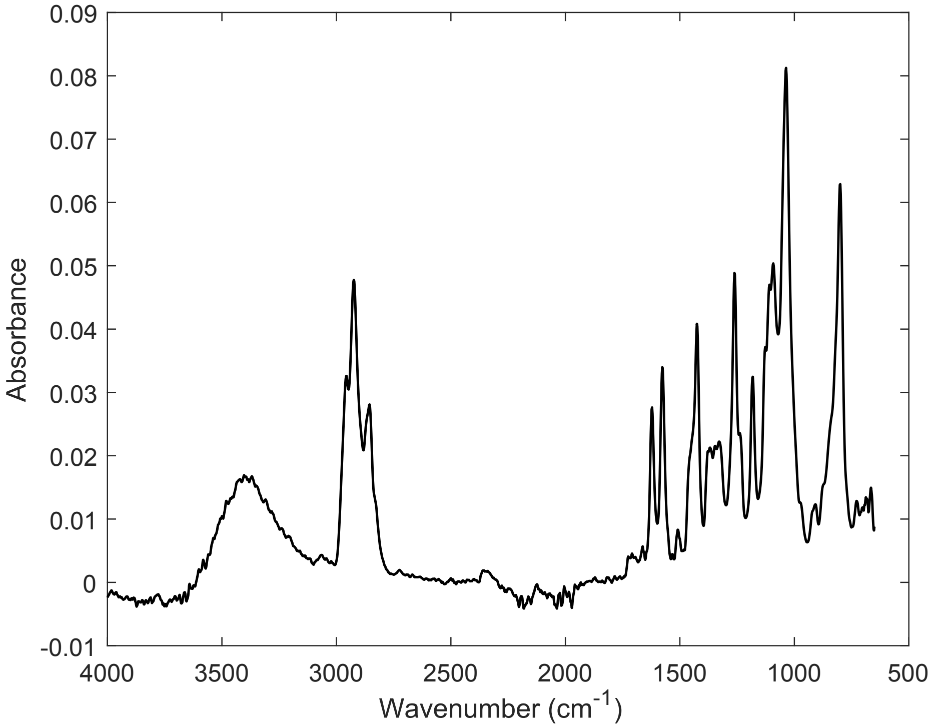

Fig. S2. Mid-infrared absorbance spectra of THC standard solution.

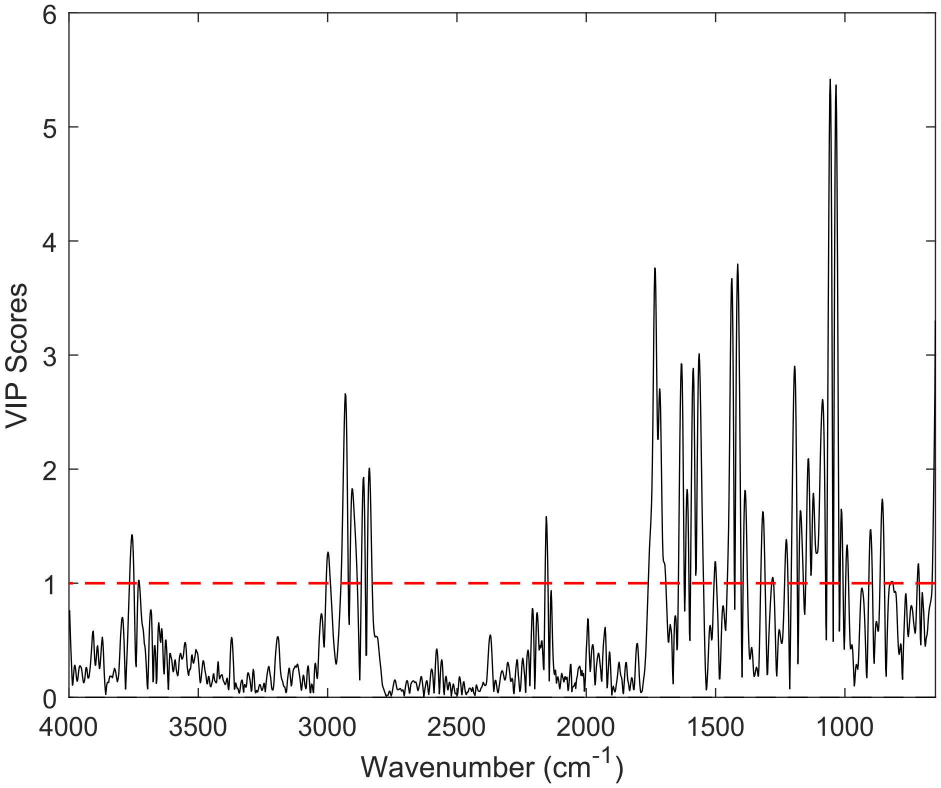

Fig. S3. VIP scores for model A.

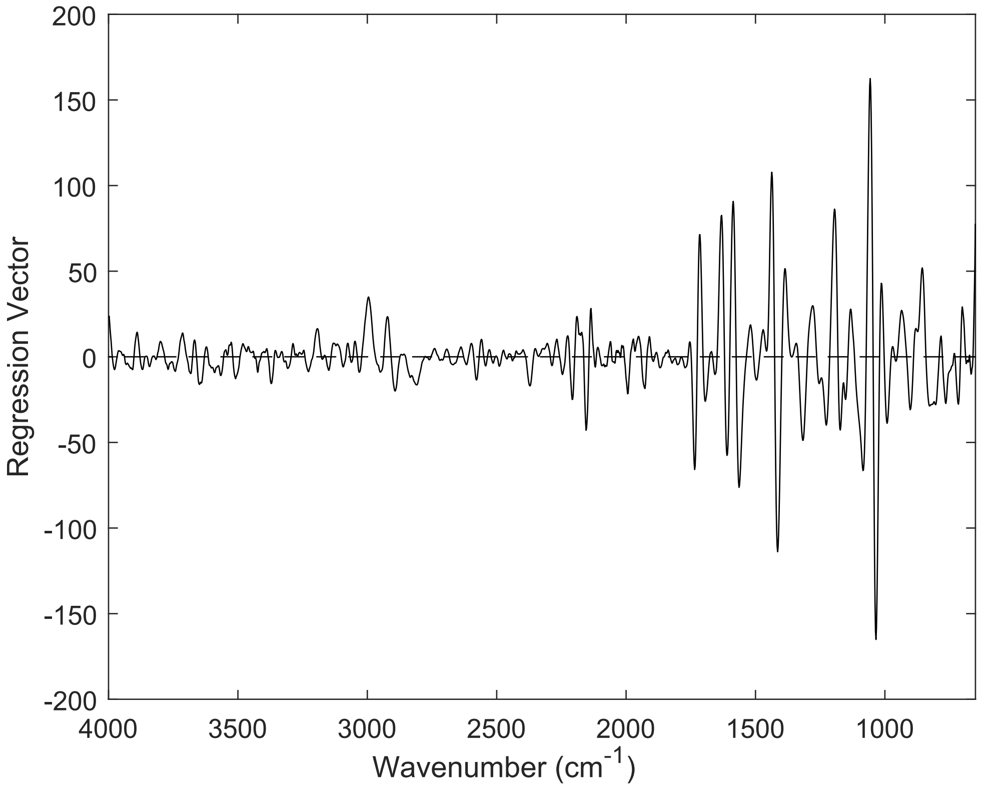

Fig. S4. Regression vector for model A.

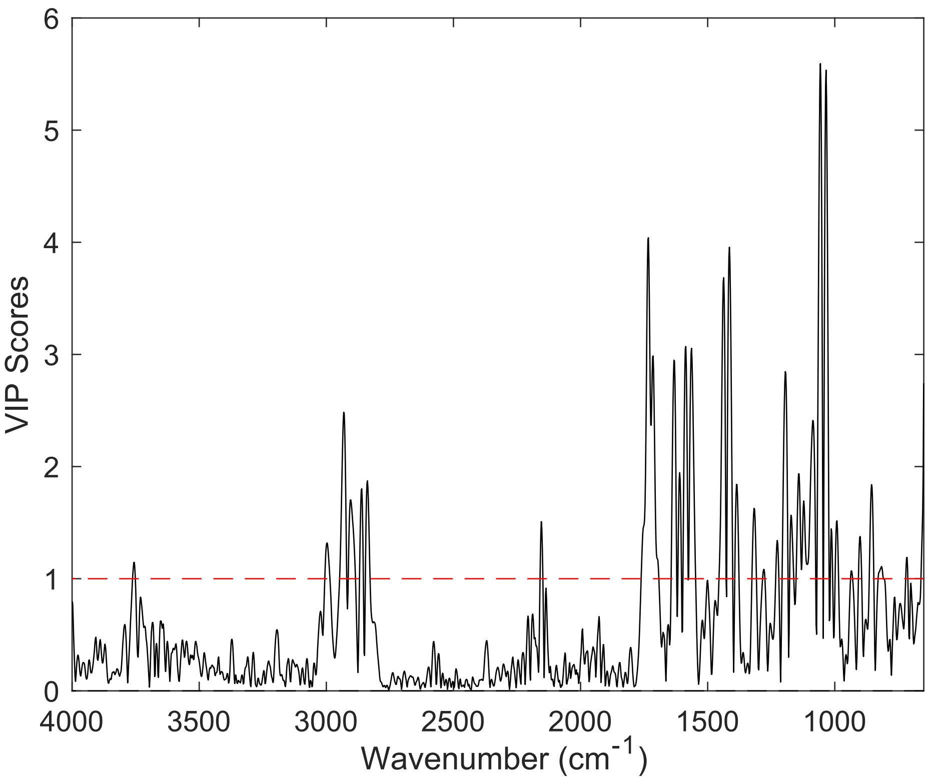

Fig. S5. VIP scores for model B.
